# An antibody against an *Anopheles albimanus* midgut myosin reduces *Plasmodium berghei* oocyst development

**DOI:** 10.1186/s13071-016-1548-8

**Published:** 2016-05-10

**Authors:** Alba N. Lecona-Valera, Dingyin Tao, Mario H. Rodríguez, Tomás López, Rhoel R. Dinglasan, María C. Rodríguez

**Affiliations:** Center of Research on Infectious Diseases, National Institute of Public Health, Av. Universidad 655, Col. Santa María Ahuacatitlán, Cuernavaca, Morelos C. P. 62508 Mexico; W. Harry Feinstone Department of Molecular Microbiology & Immunology and the Malaria Research Institute, Johns Hopkins Bloomberg School of Public Health, 615 North Wolfe Street, Baltimore, Maryland 21205 USA; Instituto de Biotecnología, Universidad Nacional Autónoma de Méxic006F, Av. Universidad 2001, Colonia Chamilpa, Cuernavaca, Morelos 62210 Mexico

**Keywords:** *Anopheles albimanus*, Midgut, Myosin, *Plasmodium berghei*, Monoclonal antibody

## Abstract

**Background:**

Malaria parasites are transmitted by *Anopheles* mosquitoes. Although several studies have identified mosquito midgut surface proteins that are putatively important for *Plasmodium* ookinete invasion, only a few have characterized these protein targets and demonstrated transmission-blocking activity. Molecular information about these proteins is essential for the development of transmission-blocking vaccines (TBV). The aim of the present study was to test three monoclonal antibodies (mAbs), A-140, A-78 and A-10, for their ability to recognize antigens and block oocyst infection of the midgut of *Anopheles albimanus*, a major malaria vector in Latin America.

**Method:**

Western-blot of mAbs on antigens from midgut brush border membrane vesicles was used to select antibodies. Three mAbs were tested by membrane feeding assays to evaluate their potential transmission-blocking activity against *Plasmodium berghei*. The cognate antigens recognized by mAbs with oocyst-reducing activity were determined by immunoprecipitation followed by liquid chromatography tandem mass spectrometry.

**Results:**

Only one mAb, A-140, significantly reduced oocyst infection intensity. Hence, its probable protein target in the *Anopheles albimanus* midgut was identified and characterized. It recognized three high-molecular mass proteins from a midgut brush border microvilli vesicle preparation. Chemical deglycosylation assays confirmed the peptide nature of the epitope recognized by mAb A-140. Immunoprecipitation followed by proteomic identification with tandem mass spectrometry revealed five proteins, presumably extracted together as a complex. Of these, AALB007909 had the highest mascot score and corresponds to a protein with a myosin head motor domain, indicating that the target of mAb A-140 is probably myosin located on the microvilli of the mosquito midgut.

**Conclusion:**

These results provide support for the participation of myosin in mosquito midgut invasion by *Plasmodium* ookinetes. The potential inclusion of this protein in the design of new multivalent vaccine strategies for blocking *Plasmodium* transmission is discussed.

**Electronic supplementary material:**

The online version of this article (doi:10.1186/s13071-016-1548-8) contains supplementary material, which is available to authorized users.

## Background

Malaria kills approximately 584,000 people annually, mainly children under five years of age [1]. *Plasmodium* parasites are the causative agents of this disease and are transmitted to humans by *Anopheles* mosquitoes. During the life cycle of *Plasmodium,* sexual stage gametocytes in humans pass to *Anopheles* mosquitoes in a blood meal. Male and female gametes then egress from their host red blood cell, and fertilized female macrogametes transform into motile ookinetes that can invade the mosquito midgut epithelium. Upon reaching the basal lamina, ookinetes develop into oocysts. Thousands of sporozoites form in mature oocysts and then escape into the hemocoel. From there they can invade the salivary glands and be inoculated into new vertebrate hosts during subsequent feeding events [2].

Without the successful development of malaria parasites in the mosquito vector midgut, parasite transmission to vertebrates is not possible [3]. Transmission-blocking vaccines (TBV), proposed as a complementary strategy to combat malaria, target either the parasite stages that develop in the mosquito midgut or their cognate midgut receptors. By interfering with the molecular interactions necessary for the fertilization of gametes, the ookinete invasion of the midgut epithelium, or the ookinete-to-oocyst transition, these TBVs could prevent malaria transmission [4] and thus serve as an important tool for malaria elimination and eradication [5].

Some molecules on the apical surface of the *An. gambiae* midgut are known to play an important role in ookinete invasion, including a conserved *O*-linked glycan on midgut glycoproteins [6], several annexin-like protein isoforms [7], aminopeptidase N1 (APN1) [8], sulfated proteoglycans [9], carboxypeptidase B (CPB) [10, 11], Croquemort scavenger receptors (SCRBQ2) [12], a secreted glycoconjugate of unknown function (SGU) [13], and the enolase binding protein (EBP) [14]. However, there is still limited information about the molecular structure of midgut proteins that are potentially unique to mosquitoes in the Americas. One such mosquito, *An. albimanus,* is a major malaria vector in Latin America [15]. The aim of the present study was to test three monoclonal antibodies (mAbs), A-78, A-10 and A-140, for their ability to recognize antigens and block oocyst infection of the midgut of *An. albimanus*. We herein report the transmission-blocking activity of monoclonal antibody (mAb) A-140 and the characterization of its probable target antigen in the midgut of *An. albimanus*.

## Methods

### Ethics statement

This study was authorized by the Ethics in Animal Care committee of the Mexican National Institute of Public Health (INSP: Instituto Nacional de Salud Pública, reference number CI:939).

### Mosquitoes

Female *An. albimanus* mosquitoes (3-5 day-old) from the white striped colony [16] at the insectary of INSP were maintained in standard rearing conditions (25 °C and 80 % humidity) and fed with cotton pads soaked in 4 % sucrose water solution. Midguts from groups of female mosquitoes were dissected in PBS supplemented with 1X complete EDTA-free protease inhibitor cocktail (Roche) and stored at -70 °C.

#### Brush border membrane vesicle (BBMV) preparation

BBMV were obtained from frozen midguts (*n* = 1,000) as described previously [17], with some modifications. Briefly, midguts were transferred to 500 μl of microvilli buffer (300 mM mannitol, 20 mM Tris-HCl, 5 mM EGTA at pH 7.4, 2 mM dithiothreitol, 0.5 mM phenylmetylsulfonyl fluoride, and 2× complete EDTA-free protease inhibitor cocktail; Roche) on ice. The midguts were homogenized by 10 cycles of 30 s each (with 30-second intervals) with a Dounce homogenizer. Four hundred and 50 μl of microvilli buffer and 50 μl of 250 mM MgCl_2_ were added and mixed by vortexing. After 20 min of incubation on ice, the sample was centrifuged at 3,500 *g* and 4 °C for 15 min. The supernatant was collected and the pellet suspended in 500 μl of microvilli buffer, then extracted twice as aforementioned. Supernatants from all extractions were pooled and subjected to ultracentrifugation at 30,000 *g* and 4 °C for 1 h. The supernatant was discarded and the pellet was suspended in 300 μl of PBS. To verify BBMV enrichment, 1 μl of the suspended pellet solution and 1 μl of the initial crude homogenate were assayed for aminopeptidase specific activity using L-leucine-p-nitroanilide as substrate [18]. Protein quantification of the BBMV was performed using a BCA protein assay Kit (Pierce, Rockford IL). The BBMV preparation was stored at -70 °C to await further use.

#### Monoclonal antibody production

*Anopheles albimanus* mosquito midguts were dissected and snap-frozen in sterile PBS containing a protease inhibitor cocktail (P8340, Sigma Chemical Co.) To produce mAbs we followed the protocol described by Niebuhr et al. [19]. Hybridoma cells were generated by fusion of cells obtained from the popliteal lymph node with PAI myeloma cells (kindly donated by Jean Langhorne, Francis Crick Institute, UK) using the polyethylene glycol method as previously described [19]. Hybridomas were selected with HAT medium and screened by Western-blot using nitrocellulose membranes containing BBMV. The selected hybridoma cells were expanded and subcloned by limiting dilution [20]. Ascitic fluid was produced for each monoclonal antibody following the methods described by Harlow & Lane [20]. Monoclonal antibodies (A-78, A-10 and A-140) were purified from ascitic fluid using Hi-Trap columns packed with Protein G Sepharose (Invitrogen). MAb isotypes were determined with a Pierce Rapid Isotyping Kit-Mouse (Thermo Scientific) according to the manufacturer’s instructions.

#### Production of immune serum against BBMV

One BALB/c mouse was immunized intraperitoneally to produce immune serum against BBMV as described [19]. Naive serum was obtained before immunization. Two weeks after the third immunization the mouse was cardiac bled and the serum stored at -20 °C. This immune serum is denominated hereafter as IS-BBMV.

#### Electrophoresis and Western blot analysis

BBMV (150 μg of protein in a preparative gel) were separated by sodium dodecyl sulfate-polyacrylamide 8 % gel electrophoresis (SDS-PAGE) [21]. A portion of the gel was cut and stained with colloidal Coomassie blue G-250, and the remaining gel was transferred to a nitrocellulose membrane [22]. The membrane was cut into strips, which were blocked with 0.05 % PBS-tween and 5 % skim milk (blocking solution) for 1 h. Individual strips were treated with undiluted conditioned medium from the hybridoma cell cultures and the IS-BBMV diluted 1:20,000 in blocking solution (included as positive control). Membranes were incubated overnight at 4 °C and treated with goat anti-mouse IgG (Abcam) conjugated to horseradish peroxidase (HRP), diluted 1:5000, at room temperature for 1 h. Bound antibodies were revealed by a chemiluminescent reaction with the Immobilon Western kit (Millipore).

#### *Plasmodium berghei* parasites

*Plasmodium berghei* parasites, constitutively expressing the green fluorescent protein (GFP, kindly provided by Robert E. Sinden, Imperial College, UK) [23], were cultured *in vitro* to produce ookinetes [24]. Parasite cultures were centrifuged at 1,000 *g* for 5 min and the concentration of ookinetes was determined and adjusted to 1,000 ookinetes/μl [25] by adding normal mouse blood (treated exactly as the aforementioned infected mouse blood) adjusted to 40 % hematocrit using heat-inactivated fetal bovine serum.

#### Membrane feeding assay with mAbs

For each experiment, groups of 100 female *An. albimanus* were fed with the GFP ookinetes (1,000 ookinetes/μl) incubated with purified mAbs A-78, A-10 or A-140 (all of which recognize the BBMV) at indicated concentrations. Four experiments were performed, in the first one, only mAb A-78, A-10, positive and negative controls were included. The positive control was fed with ookinetes plus 100 μg/ml of mAb 13.1 (anti P28, kindly donated by R. Sinden, Imperial College, UK), which significantly inhibits the transition from ookinetes to oocysts [26]. The negative control was fed with a similar number of ookinetes but without any antibody. Since neither mAb A-78 nor mAb A-10 reduced the number of oocyst formed per midgut in the following three experiments, only mAb A-78 was included as negative control. Infections were carried out by the standard Membrane Feeding Assay (MFA) [27]. To increase the possibility that all mosquitoes would take a full blood meal, they were starved for at least 5 h before being offered the ookinete preparations (maintained at 37 °C). Mosquitoes that did not feed were discarded, while engorged mosquitoes were transferred to an incubator at 21 °C, where they were maintained for 8-10 days and fed with a solution containing 8 % fructose and 0.5 % para-aminobenzoic acid [28]. Surviving mosquitoes were dissected and their midguts analyzed using a fluorescent microscope (Leica DM1000); around 30 mosquitoes per group that took a meal, survived until the end of each experiment. The percentage of infected mosquitoes (prevalence of infection) and the number of oocysts per mosquito (intensity of infection) were determined.

#### Statistical analysis

The effect of mAb A-140 was evaluated by counting the number of oocysts per midgut and the data analyzed. A zero-inflated Generalized Linear Mixed Model (GLMM) [26] was employed to compare the numbers of oocysts per mosquito midgut following infection by using the R package software.

#### Characterization of the antigens recognized by mAb A-140

To investigate if the antigen recognized by mAb A-140 was a carbohydrate residue on the proteins, chemical deglycosylation was carried out as previously described [27], with some modifications. Briefly, three nitrocellulose membrane strips that contained BBMV were washed with 50 mM sodium acetate at pH 4.5 (washing solution) at RT for 1 h. One strip was incubated with 20 mM sodium periodate in washing solution in the dark at RT for 1 h. Under these conditions, sodium periodate converts cis-diol groups (on the protein backbone of all oligosaccharides) into aldehydes, destroying the carbohydrate residue without altering protein or lipid epitopes [27]. A second strip was incubated with 1 mM sodium periodate in washing solution in the dark at 4 °C for 1 h. Under these conditions sodium periodate destroys *N*- and *O*-linked glycans. A third strip, kept in washing solution under the same conditions but without the addition of sodium periodate, was used as a control. All strips were rinsed with three changes of washing solution, incubated with 50 mM sodium borohydride in PBS at RT for 30 min, and washed three times with PBS. Finally, BBMV antigens recognized by mAb A-140 were detected by Western blot analysis. An additional control included a group of three nitrocellulose membrane strips containing BBMV that were treated as described and tested with IS-BBMV.

#### Identification of the antigens recognized by mAb A-140

To identify the proteins recognized by mAb A-140, we used an immunoprecipitation assay followed by liquid chromatography tandem mass spectrometry (LC-MS/MS). Two hundred midguts that had been frozen in PBS containing 2X protease inhibitor cocktail (Sigma) were thawed and suspended in 1 ml of cold PBS (also containing 2X protease inhibitor cocktail), and spun at 500 *g* for 1 min to gently pellet the midguts. After centrifugation the supernatant was discarded and the midgut pellets were suspended in 1 ml of cold (4 °C) solubilization buffer (50 mM Tris, 150 mM NaCl, 100 mM EDTA, 1 % NP40 and 1 % deoxycholic acid) supplemented with the 2X protease inhibitor cocktail and homogenized by using an insulin syringe to take in and expel the sample (five times with 2 min intervals) at 4 °C during 10 min. After centrifugation at 15,000 *g* for 5 min, the supernatant was discarded and the pellet (insoluble fraction) was solubilized using 75 μl of solubilization buffer plus 25 μl of loading buffer (containing 8 % SDS and 40 % glycerol). The sample was boiled for 5 min and centrifuged at 15,000 *g*. The supernatant was diluted 10-fold with cold solubilization buffer (supplemented with 2X protease inhibitor cocktail) in order to dilute the SDS to 0.2 %. A sample of 1 ml (final volume) was cleared by adding 20 μl of suspended volume of Protein G PLUS-Agarose beads (Santa Cruz), then incubated on a rocker platform at 4 °C for 30 min. Afterwards, the sample was centrifuged at 500 *g* and 4 °C for 5 min, the supernatant was transferred to a new Eppendorf tube (1.5 ml), 3 μg of mAb A-140 were added, and incubation was carried out at 4 °C for 1 h. Then 25 μl of the suspended volume of Protein G PLUS-Agarose were added, followed by incubation on a rocker platform and centrifugation at 500 *g* and 4 °C for 5 min. The pellet was suspended and washed 4 times using solubilization buffer supplemented with 2X protease inhibitor cocktail followed by centrifugation (as aforementioned). After the final wash, the supernatant was discarded and the bead pellets were suspended in 40 μl of 1X electrophoresis reducing sample buffer and boiled for 5 min. Finally, the sample was centrifuged at 500 *g* and 4 °C for 5 min and the supernatant was resolved by SDS-PAGE (8 %). The gel was visualized by colloidal Coomassie blue G-250 staining and the bands that corresponded to the molecular weight of the antigens recognized by mAb A-140 were cut from the gel for analysis by LC-MS/MS. Gel slices were cut into 1 × 1 mm pieces prior to de-staining, reduction and alkylation, tryptic digestion and peptide extraction. The extracted peptides were lyophilized and were then re-suspended in 2 % acetonitrile, 97.9 % water and 0.1 % formic acid buffer for LC-MS/MS analysis.

#### LC-MS/MS

In-gel digestion of biological replicate samples from three independent experiments were analyzed as follows. Each of the in-gel digestion samples were injected onto an Agilent LC-MS system comprised of a 1200 LC system coupled to a 6520 Q-TOF via an HPLC Chip Cube interface. The sample was trapped and analyzed using an Agilent Polaris-HR-Chip-3C18 chip (360 nL, 180 Å C18 trap with a 75 μm i.d., 150 mm length, and 180 Å C18 analytical column). Peptides were loaded onto the enrichment column automatically by the autosampler using 97 % solvent A (0.1 % formic acid in water) and 3 % solvent B (0.1 % formic acid in 90 % acetonitrile) at a flow rate of 1.8 μl/min. Elution of peptides from the analytical column was performed using a gradient starting at 97 % A at 300 nL/min. The mobile phase was 3-10 % B for 1 min, 10-40 % B for 19 min, 40-90 % for 3 min, and maintained at 90 % B for 6 min, followed by re-equilibration of the column with 3 % B for 6 min. The data dependent (autoMS2) mode was used for MS acquisition by Agilent 6520 Q-TOF in 2 GHz. Precursor MS spectra were acquired from m/z 315 to 1700 and the top 4 peaks were selected for MS/MS analysis. Product scans were acquired from m/z 50 to 1700 at a scan rate of 1.5/second. A medium isolation width (∼4 amu) was used, and a collision energy of slope 3.9 V/100 Da with a 2.9 V offset was applied for fragmentation. A dynamic exclusion list was applied, with precursors of 0.50 min excluded after the two MS/MS spectrum was acquired.

#### Database search

LC-MS/MS raw data were converted to Mascot generic Format (.mgf) by Agilent MassHunter Qualitative Analysis B.04.00. Mascot version 2.4.1 was used to search against three different FASTA databases downloaded from VectorBase including Anopheles-albimanus-AalbS1.2 (11,994 sequences), Anopheles-darlingi-AdarC3.2 (10,457 sequences), and Anopheles-gambiae-AgamP3.8 (14,667 sequences), for peptide sequence assignments using the following parameters: precursor ion mass tolerance of 50 ppm and a fragment ion mass tolerance of 0.2 Daltons. Peptides were searched using fully tryptic cleavage constraints and up to two internal cleavage sites were allowed for tryptic digestion. Variable modifications considered were carbamidomethylation of cysteine and oxidation of methionine residues. Overall, a protein false discovery rate of less than 1 % with an ion score ≥ 39 was obtained through Mascot for protein identification.

## Results

### Three monoclonal antibodies generated against whole midguts recognized apical BBMV proteins

**Fig. 1:**
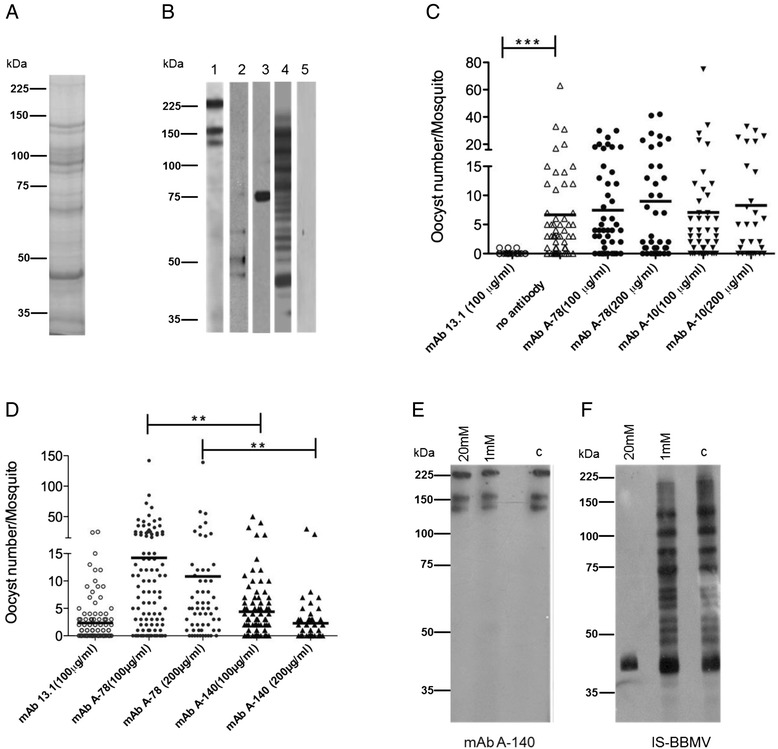
Immunoblot analysis of monoclonal antibody protein recognition profiles. **a** BBMV proteins were resolved in SDS-PAGE and stained with colloidal Coomassie blue. **b** Western blot analysis of BBMV probed with mAb A-140 (Lane 1), mAb A-78 (Lane 2), mAb A-10 (Lane 3), immune serum generated against the microvillus extract (IS-BBMV) (Lane 4), and naive serum (Lane 5). **c** MAbs A-78 and A-10 did not show a significant reduction in the intensity of infection of *P. berghei* in *An. albimanus* mosquitoes. **d** A representative pooled data is shown from replicate experiments, mAb A-140 significantly reduced the intensity of infection of mosquitoes (see Table 1 and Additional file 1 for details). **e** and **f** Reactivity of mAb A-140 and IS-BBMV following chemical deglycosylation. Membrane strips were treated with 1 or 20 mM sodium periodate, or untreated (control = C). Molecular masses are indicated in kilodaltons (kDa)

We consistently obtained around 200 μg of protein from the BBMV of 1,000 midguts. As expected, these preparations showed a 5-6 fold increase in aminopeptidase activity (data not shown) compared to whole midgut preparations [28]. One of these reproducible BBMV preparations (resolved by SDS-PAGE and stained with colloidal Coomassie blue G-250) revealed the range in protein molecular masses in this midgut fraction (Fig. 1, panel A) [29].

We tested supernatants from 27 hybridoma cell cultures by Western blot to: 1) identify hybridomas producing antibodies that can recognize BBMV, and 2) determine the repertoire of proteins in the *An. albimanus* midgut BBMV recognized by the antibodies. Only three mAbs (A-140, A-78 and A-10) recognized BBMV proteins, and each of these exhibited different protein banding profiles (Fig. 1, panel B, Lanes 1, 2 and 3). MAb A-140 (IgG1subclass) recognized three proteins bands (Fig. 1, panel B, Lane 1), one of an apparent molecular weight of 225–230 kDa and the other two of ~153 and ~136 kDa. MAb A-78 (IgG2b) recognized three bands of ~60, ~48 and ~42 kDa (Fig. 1, panel B, Lane 2). MAb A-10 (IgG2b) recognized one protein band of ~75 kDa. IS-BBMV, included as a positive control, recognized approximately 16 protein bands from the BBMV across a broad range of molecular masses (Fig. 1, panel B, Lane 3). We did not observe any protein bands below 35 kDa by any mAb after resolving BBMV proteins in 4-20 % gradient gels prior to immunoblotting (data not shown). No bands were recognized by naive serum (Fig. 1, panel B, Lane 5) or when primary mAbs were omitted (data not shown).

### MAb A-140 reduced *P. berghei* oocyst formation

Mosquito groups were fed with *P. berghei* ookinetes and one of three *An. albimanus* mAbs in order to evaluate the capacity of these antibodies to block or reduce ookinete midgut invasion. In the first experiment of MFA we observed that mosquitoes that were fed mAb A-78 and A-10 had similar oocyst intensities as compared to the negative control group, which was fed with ookinetes but without any antibody (Fig. 1c). Therefore, we chose mAb A-78 as the negative control for the three subsequent independent experiments of MFA. Oocyst intensity was significantly lower when using mAb A-140, at both 100 μg/ml (*P* < 0.0001) and 200 μg/ml (*P* < 0.0001), compared to mAb A-78 at matching concentrations (Fig. 1d, Table 1, for additional information about statistical analysis see Additional file 1). The mean number of oocysts was similar between mosquitoes fed with mAb A-140 (at 200 μg/ml) and the positive control with anti-P28 mAb13.1 (*P* = 0.324, Additional file 1).

**Table 1:** Reduction of *Plasmodium berghei* oocyst intensity in *Anopheles albimanus* by mAb A-140

Treatment	Mosquitoes fed/group	Prevalence (% infected mosquitoes)	Mean number of oocysts/mosquito (Range)	% Reduction in oocyst intensity	*P -*value
(C+) mAb 13.1 (100 μg/ml)	114	54	2.3 (0–25)	83	< 0.0001
(C-) mAb A-78 (100 μg/ml)	104	82	14.2 (0–142)	0	–
(C-) mAb A-78 (200 μg/ml)	59	83	10.8 (0–139)	0	–
mAb A-140 (100 μg/ml)	104	71	4.3 (0–50)	69.2	< 0.0001
mAb A-140 (200 μg/ml)	60	53	2.2 (0–30)	78.9	< 0.0001

Transmission reduction efficiency of mAb A-140 was evaluated at two different doses and compared to anti-P28 mAb 13.1 (positive control, C+) and mAb A-78 (negative control, C-), which was raised against *An. albimanus* midguts but was determined previously to have no functional activity against the parasite. *P-*values as determined by a zero-inflated GLMM statistical methods (see Additional file 1 for details) in comparisons with matched concentrations of mAb A-78 (100 μg/ml or 200 μg/ml). A representative dataset is shown below from replicate studies. Data correspond to Fig. 1d

### MAb A-140 recognized a peptide epitope

A-140 recognized three bands by immunoblot, which may represent a conserved glycan epitope [6] across several BBMV glycoproteins. Following treatment with 1 mM or 20 mM sodium periodate, the A-140 recognition profile remained the same (Fig. 1, panel E) and was similar to that of the control (un-treated) (Fig. 1, panel E, Lane c). These data indicate that mAb A-140 does not recognize glycans. In contrast, the IS-BBMV showed a modified protein profile (at the 20 mM concentration), as was found in the strip treated with 1 mM sodium periodate compared to the un-treated strip (Fig. 1, panel F, Lane c). This suggests that a subset of antibodies in the polyclonal response to BBMV immunization recognizes glycans.

### MAb A-140 immunoprecipitated three main protein bands from *An. albimanus* midguts

**Fig. 2:**
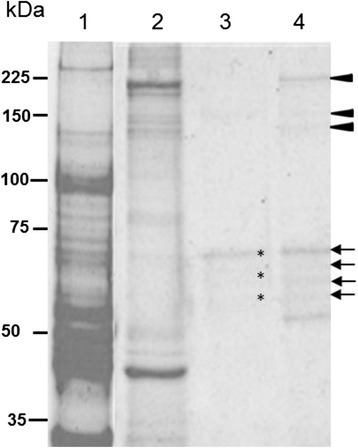
Immunoprecipitation of *An. albimanus* midgut proteins using mAb A-140. Representative fractions obtained from the immunoprecipitation of midguts using mAb A-140, analyzed by SDS-PAGE (8 %) and stained with colloidal Coomassie blue. The whole midgut was solubilized with RIPA buffer (Lane 1). The insoluble fraction was solubilized with RIPA + SDS 2 % (Lane 2). The proteins shown are those that bound to Protein G agarose during the pre-clearing step (Lane 3). The proteins shown are those that immunoprecipitated with Protein G agarose + mAb A-140 (Lane 4). Molecular masses are indicated in kDa

From three immunoprecipitations of midgut protein extracts with mAb A-140, we were able to enrich three protein bands that were visualized by Coomassie (Fig. 2, Lane 4, arrow head). These bands corresponded to the protein banding profile for mAb A-140 (Fig. 1, panel B, Lane 1) in BBMV immunoblots. Additionally, five more protein bands, between 50-70 kDa and bound to the Protein G beads, were also enriched following immunoprecipitation (Fig. 2, Lane 4, arrows). Four of these bands were also observed in the pre-cleared fraction (Fig. 2, Lane 3, asterisks), but these proteins did not correspond to any of those detected in BBMV immunoblots using mAb A-140 (Fig. 1, panel B, Lane 1).

Coomassie-stained bands (Fig. 2, Lane 4, arrowheads) were excised from the gels of each of the three replicate immunoprecipitation assays. We identified five proteins with extremely high Mascot scores (Table 2). Myosin (AALB007909) was a major component in all replicate immunoprecipitated samples. This is likely not a contaminating protein that bound non-specifically to the Protein G beads, since no high molecular mass protein was observed across replicate cleared samples. We also identified a secreted, heavily glycosylated hypothetical protein containing an insulin-like growth factor binding protein (ILGFBP) domain (AALB001083) with a predicted M_r_ of 153 kDa. Interestingly, we found three other cytosolic proteins in addition to myosin: Lingerer protein (AALB004761), Apaf-1-like killer protein (ARK) (AALB010165), and another hypothetical protein with several domains (Calcium binding domain/Kazal-type serine protease inhibitor domain/Thyroglobulin Type 1 domain) (AALB005141).

**Table 2:** *Anopheles albimanus* midgut proteins that were immunoprecipitated using mAb A-140 and identified by LC-MS/MS

Protein name	Accession No.	Mascot score	M_r_ (kDa)	Signal P*	Features
Myosin heavy chain	AALB007909-PA	6197	250	N	Myosin head motor domain
Hypothetical protein	AALB001083-PA	994	153	Y	Insulin-like growth factor binding protein domain, N-terminal domain/4 N-linked glycosylation sites*/41 O-linked glycosylation sites*
Protein lingerer	AALB004761-PA	108	154	N	Gly-Ala rich N-terminal domain/Ser-Gly-Ala-Gln rich C-terminal domain
Apaf-1 like killer protein (ARK)	AALB010165-PA	41	164	N	N-terminal death domain/P-loop Nucleoside triphosphate hydrolase/C-terminal WD-40 repeat domains
Hypothetical protein	AALB005141-PA	39	192	N	Calcium binding domain/Kazal-type serine protease inhibitor domain/Thyroglobulin Type 1 domain

*Prediction algorithms: SignalP 3.0was used to predict the presence of a signal peptide (http://www.cbs.dtu.dk/services/SignalP/). NetNGlyc 1.0 and NetOGlyc 4.0 cut-off scores (>0.7 threshold) (http://www.cbs.dtu.dk/services/). GlycoEP (http://www.imtech.res.in/raghava/glycoep/index.html) was also used to predict *N*- and *O*-linked glycan modification sites. The number of sites in common between NetNGlyc/NetOGlyc and GlycoEP are indicated in the table above

### MAb A-140 recognized protein bands from *Drosophila* and mouse skeletal muscle

**Fig. 3:**
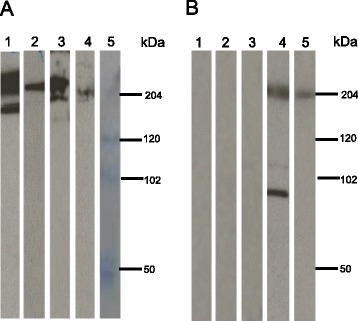
Immunoblot analysis of protein profiles of mAb A-140 and anti-myosin heavy chain mAb probed on *Drosophila* and mouse muscle. **a** Samples probed with mAb A-140. **b** Samples probed with commercial anti-myosin heavy chain mAb. Samples of *An. albimanus* midguts (Lane 1), *Drosophila* midguts (Lane 2), complete *Drosophila* (without head) (Lane 3), skeletal muscle from mouse leg (Lane 4), and the molecular mass ladder, including recombinant myosin (Lane 5). Molecular masses are indicated in kDa. Note that in Lane 5 anti-myosin heavy chain reacts with myosin, from the molecular weight standards

To confirm that mAb A-140 recognizes myosin, as opposed to a myosin-like protein, we used samples of whole (without the head) *Drosophila melanogaster*, isolated midguts and mouse leg muscle. These samples were transferred onto membranes and probed with two antibodies, purified mAb-140 and a commercial anti-myosin monoclonal antibody that reacts with myosin (heavy chain) from several mammals (humans, rabbit, mouse), zebra fish and *Drosophila* (Millipore, catalog number 05-716). Unlike what was customarily observed for *An. albimanus* midguts (Fig. 3, panel A, Lane 1), mAb A-140 recognized a prominent protein band of ~ 225–230 kDa when probed on *Drosophila* midguts (Fig. 3, panel A, Lane 2), as well as a protein smear of ~ 200–250 kDa and one band of ~ 185 kDa (Fig. 3, panel A, Lane 3) when probed on whole *Drosophila* (without the head). On mouse skeletal muscle, mAb A-140 recognized (albeit weakly) one protein band of ~ 204 kDa (Fig. 3, panel A, lane 4), while the commercial anti-myosin unexpectedly did not recognize *Drosophila* or *An. albimanus* midguts. However, the latter mAb recognized three protein bands of ~ 204, 110 and 95 kDa (Fig. 3, panel B, Lane 4) and recombinant myosin (BIO-RAD molecular weight standards) (Fig. 3, panel B, Lane 5).

## Discussion

We herein report the partial characterization of a mosquito midgut molecule that seems to participate in the process of *P. berghei* ookinete infection of *An. albimanus*. This molecule was recognized by mAb A-140, which of the monoclonal antibody tested (including A-10, A-78 and A-140) was the only one capable of reducing midgut oocyst intensity. These results add new information to previous studies [6–8, 11, 30–34] using different parasite/mosquito combinations (including rodent and human malaria parasites) on the effect of monoclonal and polyclonal antibodies against midgut molecules participating in midgut invasion.

MAb A-140 was generated from a mouse immunized with “whole midgut” lysate, and was selected by Western blot using BBMV. While mAb A-140 recognized proteins in the *Drosophila* midgut and carcass (without the head) as well as in mouse skeletal muscle (Fig. 3), it failed to recognize recombinant myosin. The Western blot recognition by mAb A-140 of three high molecular mass protein bands in BBMV preparations confirmed the presence of the cognate antigens on the apical membrane.

The recognition by mAb A-140 of more than one protein band in BBMV suggested that it recognized carbohydrate isoforms of a single glycoprotein species. While periodate oxidation of glycans affects carbohydrate antigenicity (without affecting the lipids or proteins) (30), our experiments clearly demonstrated that mAb-A140 recognized peptidic epitopes. Since the BBMV sample was resolved and transferred to a nitrocellulose membrane prior to periodate treatment, a single polypeptide target with varying degrees of glycosylation [6] would nonetheless appear as a ladder of bands [35]. Alternatively, the epitope recognized by mAb A-140 could exist in more than one glycoprotein [6].

Among the five immunoprecipitated proteins, it was surprising to find AALB007909 because it corresponds to the myosin heavy chain. However, this protein has been previously observed in other insect cells. Immunoprecipitation of cell surface cadherin from *Drosophila* oocyte membranes co-precipitates myosin along with other cytoskeletal proteins and inner leaflet adaptor proteins that can bridge extracellular proteins through their transmembrane and/or cytoplasmic tails [36]. Taken together, these data indicate that the antigen of mAb A-140 is myosin, localized to the BBMV of the midgut, and that other midgut proteins co-precipitate with it. This conclusion is supported by the fact that myosin was present in all the different excised gel bands spanning the high molecular mass range proteins on an 8 % Tris-Glycine gel, and that this protein obtained the highest mascot score with LC-MS/MS.

The presence of several bands recognized by mAb A-140 (detected by Western blot) could be interpreted as cross reactivity with several myosin subtypes. It is possible that mAb A-140 recognizes an epitope present in type II myosin, which includes muscular and non-muscular forms. At present, 15 paralogues of myosin in *An. albimanus* are annotated in VectorBase [37]. The multiple band recognition by mAb may also be due to degradation products of the 250 kDa myosin (AALB007909).

The inhibitory effect of antibodies may result from a direct interference of the interaction between ookinetes and molecules on the mosquito midgut microvilli surface, as has been described by others [7, 8, 10, 34]. This interference may affect the normal physiology of both the midgut and the parasite [38, 39] or alternatively, by blocking attachment of the ookinete to the receptors present in the midgut glycocalyx or on the microvilli/plasma membrane [8, 9, 13, 40]. Myosin recognized by mAb may be involved in mediating individual membrane component associations, in accordance with the membrane fluid mosaic model [41], or in mediating clusters of these proteins that are resident on lipid rafts [42]; processes that can directly affect ookinete attachment and entry.

Non-muscle myosin expressed on the cell surface has also been shown to promote cell-virus interactions in the context of microbial pathogenesis. Non-muscle myosin heavy chain IIA (NMHC-IIA) and heavy chain II B (NMHC-IIB) function as receptors for herpes simplex virus-1 (HSV-1) through their interaction with virus glycoproteins B and D [43, 44]. Furthermore, NMHC-IIA, which is densely aggregated on the surface of nasopharyngeal epithelial cells, mediates the infection of these cells by Epstein-Barr virus [45]. In a similar manner, we propose that non-muscle myosin expressed in the midgut microvilli could be used by ookinetes as an unconventional receptor during the invasion of the midgut.

MAb A-140 binding to myosin may also be indirectly impacting midgut cell signaling after invasion [46]. Another explanation for the inhibitory effect of the antibody is that once the parasite has penetrated the midgut cell (s) they become more permeable [34] allowing mAb A-140 to bind to myosin; subsequently inhibiting molecular events required for parasite traversal of the cell. Finally, it has been reported that antibodies that affect parasite development could pass through the midgut epithelium to reach the basal labyrinth [47]. We did not test this possibility but it is likely that if the antibodies do make it to the basal labyrinth that the concentration will be drastically reduced; resulting in poor transmission-blocking activity.

Another protein immunoprecipitated by mAb A-140 was ILGFBP glycoprotein (AALB001083), which is predicted to have at least 4 *N*-linked glycosylation sites and at least 40 *O*-linked glycan modifications. High mannose and complex *N*-glycans alone are a major class of glycan posttranslational modifications in mosquitoes [48]. The presence of 40 and possibly more mucin-type *O*-glycans can increase the apparent molecular mass, as determined by SDS-PAGE, by an additional 27-30 %. This could explain the difference between the predicted M_r_ of 153 kDa and the apparent M_r_ of 225-230 kDa by SDS-PAGE and Western blot (Fig. 1, panel B, Lane 1), a mass range that is not very well resolved by 8 % Tris-Glycine gels. Additionally, the ILGFBP-related protein has a signal peptide, but it does not have a predicted transmembrane anchor or cytoplasmic tail.

The co-precipitation of three cytoplasmic proteins, ARK (AALB010165), protein lingerer (AALB004761) and a putative calcium-binding hypothetical protein (AALB005141), may indicate the interaction of these molecules with myosin and the cytoskeleton, along with the ILGFBP-related protein. ARK is the orthologue of *D. melanogaster* DARK, which assembles the apoptosome; a component of the intrinsic cell death pathway. ARK contains multiple WD repeats in the COOH-terminal region. The WD domains are involved in protein–protein interactions [49–51]. In the *Aedes aegypti* mosquito, the silencing of *ark* inhibits apoptosis triggered by several stimuli [52]. The lingerer protein from *D. melanogaster* regulates growth and interacts with several RNA binding proteins in a complex proposed to regulate translation and/or RNA stability [53]. The putative hypothetical protein (AALB005141) has several domains (Table 2) and is predicted to have the capacity to bind to calcium ions and proteins.

No plausible function of these proteins during midgut invasion by *Plasmodium* ookinetes has been identified. It is likely that the co-precipitation of these proteins was the result of the multiple protein binding domains present on lingerer and ARK which ectopically bind to ILGFBP-related protein and myosin after midgut lysis and solubilization, prior to immunoprecipitation. It is also important to note that immunoprecipitation of target proteins from a cell lysate leaves open the possibility that ectopic protein-protein interactions would result in an immunoprecipitated protein repertoire, which would include artefacts (as opposed to contaminants) following pull-down.

There is diversity in parasite-midgut interactions. Some molecules can play major roles, while others may represent additional/alternative mechanisms or members of a larger complex. Thus, antibodies for a molecule (anti-AnAPN1) conserved among several species of anopheline mosquitoes [29], even at low concentrations, have been shown to completely inhibit *P. falciparum* and *P. vivax* mosquito infection [54, 55]. Another major difference in the pathways used by *Plasmodium* parasites during invasion is exemplified by *P. berghei* ANKA ookinetes subclones that exhibit differential use of the Enolase Binding Protein, indicating that this parasite uses more than one ligand to invade the mosquito midgut [14].

Thus, the incomplete blockade of mosquito infection achieved by mAb A-140 may reflect its recognition of an alternative molecule (non-muscle myosin) for invasion. Additional research is required to identify the specific epitope recognized by this antibody in myosin. It is also important to identify its cognate ookinete surface or secreted protein ligand and to delineate the mechanism of their involvement in vector host-parasite protein interactions during midgut invasion.

It is paramount to characterize molecules from the parasite and the mosquito midgut that play a key role in the mechanisms of midgut invasion as well as the development of ookinetes into oocysts, since both events are essential for parasitic infection and malaria transmission. In-depth knowledge of such molecules is a prerequisite for the development of multivalent mosquito-based transmission-blocking vaccines, which is part of a promising strategy for malaria elimination. *Plasmodium berghei*/*An. gambiae* models have allowed for the identification of several mosquito target molecules that play a role in midgut invasion [7, 9, 10]. Nevertheless*,* care should be taken when extrapolating murine malaria data to human malaria biology [8, 13, 14]. Similar to AnAPN1 [54], the orthologs of AALB007909 are conserved across the major anopheline vectors of malaria parasites. On the other hand, although our results provide indications of the participation of myosin in *Plasmodium* ookinete invasion of mosquito midgut cells, mAb A-140 also recognized a molecular weight protein similar of that of mouse myosin, raising the possibility of generating cross reactivity to human myosin. In order to include a molecule in vaccine design is essential to avoid cross reactivity with the host’s molecules. As the heavy chain of myosin are relatively conserved in all myosin [56] it will be possible that a vaccine containing mosquito myosin as immunogen will induce myositis [57]. In this sense, more studies are needed in order to determine if specific epitopes of anopheline myosin exist that are involved in *Plasmodium* invasion of the midgut, only in this sense, the inclusion of myosin orthologs could be proposed as vaccine candidates.

## Conclusion

In this work we report on a mAb A-140, which recognizes a protein localized in the brush border membrane vesicles of *Anopheles albimanus* midgut that can significantly decrease *P. berghei* oocyst intensity. We found that mAb A-140 immunoprecipitated a complex of five proteins that was resistant to detergent solubilization from a lysate of whole mosquito midguts. The most abundant protein identified in this complex by LC-MS/MS was myosin. The antibody also recognized other non-myosin proteins with molecular weight similar to myosin from *Drosophila*. These results provide support for the unconventional involvement of myosin during mosquito midgut invasion by *Plasmodium* ookinetes and warrants further molecular dissection of this transmission-reducing activity.

## Additional file

Additional file 1: Statistical analysis GLMM (data corresponding to Fig. 1d and Table 1). (DOCX 39 kb)
