# Supplementary material for: An antibody against an Anopheles albimanus midgut myosin reduces Plasmodium berghei oocyst development
Source: Parasit Vectors. 2016 May 10;9:274. doi: 10.1186/s13071-016-1548-8 (PMC4863318; doi:10.1186/s13071-016-1548-8)
Supplement: Additional file 1: — Statistical analysis GLMM (data corresponding to Fig. 1d and Table 1). (DOCX 39 kb) [file 13071_2016_1548_MOESM1_ESM.docx]

**Additional file 1.**

**Statistical analysis GLMM (data corresponding to figure 1D and Table 1).**

|  | **mAb 13.1 (100 μg/ml)** | **A-78 (100 μg/ml)** | **A-78 (200 μg/ml)** | **A-140 (100 μg/ml)** | **A-140 (200 μg/ml)** |
| --- | --- | --- | --- | --- | --- |
| **N** | 113 | 103 | 58 | 58 | 59 |
| **# not infected** | 52 | 19 | 10 | 23 | 28 |
| **Prevalence (%)** | 54 | 82 | 83 | 60 | 53 |
| **Median** | 1.0 | 8.0 | 5.0 | 2.0 | 1.0 |
| **Mean** | 2.4 | 14.2 | 10.8 | 4.6 | 2.3 |
| **Range** | 0-25 | 0-142 | 0-139 | 0-50 | 0-30 |
| **Reduction in Mean Oocyst Intensity (%)** | 83.5 | N/A | N/A | 67.3 | 0.0 |
| **P-value (vs. mAb 13.1)** | N/A | 0.00E+00 | 3.4E-08 | 3.7E-03 | 0.3 |
| **P-value (vs. A-78-100 μg/ml)** | N/A | N/A | N/A | 4.6E-08 | N/A |
| **P-value (vs. A-78-200 μg/ml)** | N/A | N/A | N/A | N/A | 9.49E-07 |
| **Reduction in Prevalence (%)** | 33.8 | N/A | N/A | 26.0 | 36.5 |
|  |  |  |  |  |  |
|  |  |  |  |  |  |
| **Midgut** | **# Oocysts** | **# Oocysts** | **# Oocysts** | **# Oocysts** | **# Oocysts** |
| **1** | 1 | 10 | 20 | 0 | 0 |
| **2** | 1 | 28 | 5 | 14 | 0 |
| **3** | 7 | 7 | 22 | 0 | 0 |
| **4** | 3 | 23 | 55 | 0 | 0 |
| **5** | 2 | 11 | 8 | 40 | 30 |
| **6** | 7 | 65 | 1 | 2 | 0 |
| **7** | 8 | 0 | 0 | 0 | 0 |
| **8** | 0 | 7 | 2 | 0 | 1 |
| **9** | 3 | 20 | 8 | 0 | 0 |
| **10** | 0 | 3 | 13 | 1 | 0 |
| **11** | 1 | 25 | 7 | 20 | 5 |
| **12** | 25 | 3 | 58 | 5 | 0 |
| **13** | 3 | 3 | 0 | 50 | 7 |
| **14** | 10 | 35 | 3 | 6 | 0 |
| **15** | 3 | 0 | 40 | 5 | 5 |
| **16** | 1 | 8 | 0 | 0 | 1 |
| **17** | 1 | 35 | 10 | 0 | 0 |
| **18** | 1 | 12 | 0 | 0 | 3 |
| **19** | 2 | 20 | 2 | 0 | 0 |
| **20** | 3 | 0 | 1 | 1 | 3 |
| **21** | 9 | 35 | 12 | 0 | 6 |
| **22** | 0 | 5 | 33 | 1 | 0 |
| **23** | 12 | 5 | 2 | 2 | 0 |
| **24** | 4 | 10 | 0 | 6 | 1 |
| **25** | 2 | 25 | 26 | 0 | 1 |
| **26** | 1 | 15 | 1 | 0 | 0 |
| **27** | 6 | 15 | 5 | 0 | 0 |
| **28** | 0 | 45 | 0 | 3 | 0 |
| **29** | 15 | 1 | 0 | 0 | 1 |
| **30** | 0 | 2 | 0 | 0 | 2 |
| **31** | 2 | 14 | 8 | 9 | 3 |
| **32** | 1 | 0 | 139 | 5 | 0 |
| **33** | 5 | 14 | 13 | 4 | 1 |
| **34** | 0 | 13 | 6 | 0 | 8 |
| **35** | 2 | 4 | 0 | 0 | 3 |
| **36** | 1 | 10 | 3 | 2 | 0 |
| **37** | 1 | 3 | 4 | 11 | 2 |
| **38** | 0 | 5 | 3 | 1 | 1 |
| **39** | 2 | 12 | 5 | 2 | 2 |
| **40** | 1 | 1 | 10 | 2 | 0 |
| **41** | 5 | 23 | 2 | 7 | 3 |
| **42** | 3 | 0 | 4 | 2 | 0 |
| **43** | 0 | 0 | 7 | 0 | 0 |
| **44** | 1 | 11 | 10 | 0 | 2 |
| **45** | 0 | 11 | 1 | 3 | 0 |
| **46** | 0 | 32 | 1 | 4 | 0 |
| **47** | 0 | 1 | 6 | 0 | 0 |
| **48** | 0 | 142 | 18 | 2 | 0 |
| **49** | 4 | 25 | 5 | 7 | 0 |
| **50** | 0 | 0 | 5 | 5 | 3 |
| **51** | 0 | 12 | 4 | 4 | 2 |
| **52** | 0 | 0 | 5 | 20 | 7 |
| **53** | 0 | 2 | 11 | 1 | 2 |
| **54** | 0 | 0 | 1 | 3 | 21 |
| **55** | 3 | 1 | 4 | 8 | 3 |
| **56** | 0 | 21 | 6 | 10 | 1 |
| **57** | 0 | 0 | 0 | 0 | 2 |
| **58** | 0 | 42 | 12 | 0 | 4 |
| **59** | 0 | 0 | 12 | 6 | 1 |
| **60** | 1 | 21 |  |  | 0 |
| **61** | 9 | 18 |  |  |  |
| **62** | 1 | 37 |  |  |  |
| **63** | 0 | 0 |  |  |  |
| **64** | 0 | 14 |  |  |  |
| **65** | 0 | 12 |  |  |  |
| **66** | 0 | 24 |  |  |  |
| **67** | 0 | 0 |  |  |  |
| **68** | 0 | 46 |  |  |  |
| **69** | 0 | 1 |  |  |  |
| **70** | 1 | 3 |  |  |  |
| **71** | 0 | 1 |  |  |  |
| **72** | 0 | 72 |  |  |  |
| **73** | 1 | 52 |  |  |  |
| **74** | 4 | 85 |  |  |  |
| **75** | 0 | 25 |  |  |  |
| **76** | 0 | 43 |  |  |  |
| **77** | 0 | 15 |  |  |  |
| **78** | 0 | 0 |  |  |  |
| **79** | 24 | 2 |  |  |  |
| **80** | 4 | 8 |  |  |  |
| **81** | 0 | 2 |  |  |  |
| **82** | 1 | 6 |  |  |  |
| **83** | 0 | 4 |  |  |  |
| **84** | 0 | 9 |  |  |  |
| **85** | 0 | 0 |  |  |  |
| **86** | 0 | 6 |  |  |  |
| **87** | 0 | 4 |  |  |  |
| **88** | 3 | 2 |  |  |  |
| **89** | 3 | 6 |  |  |  |
| **90** | 0 | 0 |  |  |  |
| **91** | 0 | 0 |  |  |  |
| **92** | 2 | 3 |  |  |  |
| **93** | 9 | 0 |  |  |  |
| **94** | 0 | 8 |  |  |  |
| **95** | 1 | 16 |  |  |  |
| **96** | 2 | 3 |  |  |  |
| **97** | 0 | 4 |  |  |  |
| **98** | 0 | 11 |  |  |  |
| **99** | 2 | 0 |  |  |  |
| **100** | 2 | 21 |  |  |  |
| **101** | 13 | 8 |  |  |  |
| **102** | 4 | 1 |  |  |  |
| **103** | 0 | 1 |  |  |  |
| **104** | 0 | 13 |  |  |  |
| **105** | 0 |  |  |  |  |
| **106** | 0 |  |  |  |  |
| **107** | 0 |  |  |  |  |
| **108** | 2 |  |  |  |  |
| **109** | 0 |  |  |  |  |
| **110** | 1 |  |  |  |  |
| **111** | 1 |  |  |  |  |
| **112** | 12 |  |  |  |  |
| **113** | 3 |  |  |  |  |
| **114** | 0 |  |  |  |  |
|  |  |  |  |  |  |

|  |  |  |  |  |
| --- | --- | --- | --- | --- |
| **mAb 13.1 (100 μg/ml) vs A-78 (100 μg/ml)** | | | | |
|  | **Estimate** | **Std. Error** | **Z - value** | **P - value** |
| **mAb 13.1 (Intercept)** | 0.848 | 0.237 | 3.59 | 3.40E-04 |
| **A-78 (100 μg/ml)** | 1.749 | 0.22 | 7.97 | 2.00E-15 |
| P-value (difference btn mAb 13.1 and A-78) = 0 | | | | |
|  | | | | |
| **Random Effect Variance(s)** | | | | |
| **Variance** = 0.0536 | | | | |
| **Std. Dev** = 0.2315 | | | | |
| **Negative binomial dispersion parameter =** 0.50416 (std. err. = 0.14014) | | | | |
| **Zero-inflation =** 0.015138 (std. err. = 0.10588) | | | | |
|  |  |  |  |  |
|  |  |  |  |  |
| **Confidence Interval (95%)** | | | | |
|  | **2.5%** | **97.5%** |  |  |
| **mAb 13.1 (Intercept)** | 0.3844033 | 1.311545 |  |  |
| **A-78 (100 μg/ml)** | 1.3170177 | 2.180186 |  |  |
|  |  |  |  |  |
| **Output Efficacy** = -474.656 |  |  |  |  |
| **Lower Confidence Bound** = -784.795 |  |  |  |  |
| **Upper Confidence Bound** = -273.227 |  |  |  |  |
|  |  |  |  |  |
| **AIC** = 1197.1 |  |  |  |  |
| **Log-likelihood** = -593.56 |  |  |  |  |

|  |  |  |  |  |
| --- | --- | --- | --- | --- |
| **mAb 13.1 (100 μg/ml) vs A-140 (100 μg/ml)** | | | | |
|  | **Estimate** | **Std. Error** | **Z - value** | **P - value** |
| **mAb 13.1 (Intercept)** | 0.855 | 0.149 | 5.72 | 1.10E-08 |
| **A-140 (100 μg/ml)** | 0.635 | 0.212 | 2.94 | 3.30E-03 |
| P-value (difference btn mAb 13.1 and A-140) = 0.003723425 | | | | |
|  | | | | |
| **Random Effect Variance(s)** | | | | |
| **Variance** = 3.164E-09 | | | | |
| **Std. Dev** = 5.625E-05 | | | | |
| **Negative binomial dispersion parameter = 0**.47261 (std. err. = 0.05969) | | | | |
| **Zero-inflation =** 1.0E-06 (std. err. = 8.837E-07) | | | | |
|  |  |  |  |  |
| **Confidence Interval (95%)** | | | | |
|  | **2.5%** | **97.5%** |  |  |
| **mAb 13.1 (Intercept)** | 0.5619468 | 1.147623 |  |  |
| **A-140 (100 μg/ml)** | 0.2080111 | 1.038605 |  |  |
|  |  |  |  |  |
| **Output Efficacy** = 99.937 |  |  |  |  |
| **Lower Confidence Bound** = 99.81 |  |  |  |  |
| **Upper Confidence Bound** = 99.979 |  |  |  |  |
|  |  |  |  |  |
| **AIC** = 977.2 |  |  |  |  |
| **Log-likelihood** = -483.619 |  |  |  |  |

|  |  |  |  |  |
| --- | --- | --- | --- | --- |
| **A-78 (100 μg/ml) vs A-140 (100 μg/ml)** | | | | |
|  | **Estimate** | **Std. Error** | **Z - value** | **P - value** |
| **A-78 (100 μg/ml) -Intercept** | 2.66 | 0.22 | 12.05 | 2.00E-16 |
| **A-140 (100 μg/ml)** | -1.09 | 0.19 | -5.74 | 9.20E-09 |
| P-value (difference btn mAb A-78 and A-140) = 4.59E-08 | | | | |
|  | | | | |
| **Random Effect Variance(s)** | | | | |
| **Variance** = 0.08728 | | | | |
| **Std. Dev** = 0.2954 | | | | |
| **Negative binomial dispersion parameter = 0**.72779 (std. err. = 0.16227) | | | | |
| **Zero-inflation =** 0.091121 (std. err. = 0.06041) | | | | |
|  |  |  |  |  |
| **Confidence Interval (95%)** | | | | |
|  | **2.5%** | **97.5%** |  |  |
| **A-78 (100 μg/ml)-Intercept** | 2.223718 | 3.0875529 |  |  |
| **A-140 (100 μg/ml)** | -1.46441 | -0.7193105 |  |  |
|  |  |  |  |  |
| **Output Efficacy** = 66.441 |  |  |  |  |
| **Lower Confidence Bound** = 51.291 |  |  |  |  |
| **Upper Confidence Bound** = 76.879 |  |  |  |  |
|  |  |  |  |  |
| **AIC** = 1277.1 |  |  |  |  |
| **Log-likelihood** = -633.563 |  |  |  |  |

|  |  |  |  |  |
| --- | --- | --- | --- | --- |
| **mAb 13.1 (100 μg/ml) vs A-78 (200 μg/ml)** | | | | |
|  | **Estimate** | **Std. Error** | **Z - value** | **P - value** |
| **mAb 13.1 (Intercept)** | 0.479 | 0.213 | 2.24 | 2.50E-02 |
| **A-78 (200 μg/ml)** | 1.903 | 0.301 | 6.32 | 2.00E-10 |
| P-value (difference btn mAb 13.1 and A-78) = 3.35E-08 | | | | |
|  | | | | |
| **Random Effect Variance(s)** | | | | |
| **Variance** = 2.061E-09 | | | | |
| **Std. Dev** = 4.54E-05 | | | | |
| **Negative binomial dispersion parameter =** 0.38975 (std. err. = 0.062829) | | | | |
| **Zero-inflation =** 1.0E-06 (std. err. = 4.3712E-08) | | | | |
|  |  |  |  |  |
|  |  |  |  |  |
| **Confidence Interval (95%)** | | | | |
|  | **2.5%** | **97.5%** |  |  |
| **mAb 13.1 (Intercept)** | 0.06050835 | 0.8972562 |  |  |
| **A-78 (200 μg/ml)** | 1.31315619 | 2.4937209 |  |  |
|  |  |  |  |  |
| **Output Efficacy** = -570.892 |  |  |  |  |
| **Lower Confidence Bound** = -1110.624 |  |  |  |  |
| **Upper Confidence Bound** = -271.789 |  |  |  |  |
|  |  |  |  |  |
| **AIC** = 620.7 |  |  |  |  |
| **Log-likelihood** = -305.637 |  |  |  |  |
|  |  |  |  |  |
|  |  |  |  |  |
| **mAb 13.1 (100 μg/ml) vs A-140 (200 μg/ml)** | | | | |
|  | **Estimate** | **Std. Error** | **Z - value** | **P - value** |
| **mAb 13.1 (Intercept)** | 0.479 | 0.242 | 1.98 | 0.047 |
| **A-140 (200 μg/ml)** | 0.347 | 0.351 | 0.99 | 0.32 |
| P-value (difference btn mAb 13.1 and A-140) = 0.3241819 | | | | |
|  | | | | |
| **Random Effect Variance(s)** | | | | |
| **Variance** = 2.78E-07 | | | | |
| **Std. Dev** = 0.0005273 | | | | |
| **Negative binomial dispersion parameter =** 0.28923 (std. err. = 0.054384) | | | | |
| **Zero-inflation =** 1E-06 (std. err. = 1.0119E-07) | | | | |
|  |  |  |  |  |
| **Confidence Interval (95%)** | | | | |
|  | **2.5%** | **97.5%** |  |  |
| **mAb 13.1 (Intercept)** | 0.005556768 | 0.9522194 |  |  |
| **A-140 (200 μg/ml)** | -0.341595384 | 1.0350833 |  |  |
|  |  |  |  |  |
| **Output Efficacy** = -41.445 |  |  |  |  |
| **Lower Confidence Bound** = -181.534 |  |  |  |  |
| **Upper Confidence Bound** = 28.936 |  |  |  |  |
|  |  |  |  |  |
| **AIC** = 415.4 |  |  |  |  |
| **Log-likelihood** = -220.718 |  |  |  |  |
|  |  |  |  |  |
|  |  |  |  |  |
| **A-78 (200 μg/ml) vs A-140 (200 μg/ml)** | | | | |
|  | **Estimate** | **Std. Error** | **Z - value** | **P - value** |
| **A-78 (200 μg/ml)-Intercept** | 2.368 | 0.23 | 10.29 | 2.00E-16 |
| **A-140 (200 μg/ml)** | -1.546 | 0.295 | -5.24 | 0.00 |
| P-value (difference btn mAb A-78 and A-140) = 9.485E-07 | | | | |
|  | | | | |
| **Random Effect Variance(s)** | | | | |
| **Variance** = 0.006863 | | | | |
| **Std. Dev** = 0.08284 | | | | |
| **Negative binomial dispersion parameter =** 0.47447 (std. err. = 0.074968) | | | | |
| **Zero-inflation =** 1E-06 (std. err. = 8.9732E-08) | | | | |
|  |  |  |  |  |
| **Confidence Interval (95%)** | | | | |
|  | **2.5%** | **97.5%** |  |  |
| **A-78 (200 μg/ml)-Intercept** | 1.916725 | 2.8190925 |  |  |
| **A-140 (200 μg/ml)** | -2.124269 | -0.9671846 |  |  |
|  |  |  |  |  |
| **Output Efficacy** = 78.684 |  |  |  |  |
| **Lower Confidence Bound** = 61.985 |  |  |  |  |
| **Upper Confidence Bound** = 88.048 |  |  |  |  |
|  |  |  |  |  |
| **AIC** = 613.3 |  |  |  |  |
| **Log-likelihood** = -310.66 |  |  |  |  |
